# Immunomodulatory Effects of Colistin on Macrophages in Rats by Activating the p38/MAPK Pathway

**DOI:** 10.3389/fphar.2019.00729

**Published:** 2019-06-26

**Authors:** Jin Wang, Weili Shao, Hui Niu, Tianli Yang, Yuning Wang, Yun Cai

**Affiliations:** ^1^Center of Medicine Clinical Research, Department of Pharmacy, PLA General Hospital, Beijing, China; ^2^Clinical Surgery Division, PLA General Hospital, Beijing, China

**Keywords:** colistin, immunomodulatory effects, macrophages, cytokines, mitogen-activated protein kinase

## Abstract

**Objectives:** Colistin has been identified in a *Caenorhabditis elegans* chemical screening as an immunostimulatory agent that activates the conserved p38/PMK-1 pathway and provides protection against pathogens. Here we aimed to extend those findings to a mammalian model and evaluate the immunomodulatory effects of colistin on rat macrophages.

**Methods:** Macrophages were isolated from Sprague-Dawley (SD) rat. The effects of colistin on the cytokine secretion, phagocytic activity and protein expression were determined by enzyme-linked immunosorbent assay (ELISA), flow cytometry, and Western blotting analysis, respectively. The relative microRNA expression was determined by microarray, and Kyoto Encylopedia of Genes and Genomes (KEGG) was used to identify potential signaling pathways.

**Results:** Our data showed that 5, 10, and 20 µg/ml colistin significantly increased the secretion of TNF-α, while 20 and 5 µg/ml colistin significantly increased the levels of IL-1β and IL-6, respectively. Flow cytometry results showed that the relative mean fluorescence intensity and percentage of phagocytosis in colistin treatment groups were significantly higher compared with the control group, while the increased phagocytosis phenomenon can be blocked by p38 inhibitor. The phagocytic ability of macrophages against *Staphylococcus aureus* was significantly increased after colistin treatment. Microarray and KEGG pathway analyses revealed that mitogen-activated protein kinase (MAPK), mammalian target of rapamycin (mTOR), chemokine, and B cell receptor were the main pathways involved in the colistin stimulation process. Western blotting analysis demonstrated that the phosphorylated p38 protein level of colistin treatment groups was increased in a dose dependent manner.

**Conclusions:** Present study is the first to demonstrate that colistin had immunomodulatory effects on macrophages in mammals, and the p38/MAPK pathway was involved in such colistin-induced immunomodulatory effect.

Nowadays, anti-infective therapy most relies on effective antimicrobial drugs. However, increasing emergence of antimicrobial resistance has become a serious public health threat. Globally, it is conservatively estimated that more than 700,000 persons die annually due to infections caused by multidrug resistant (MDR) pathogens (Boucher et al., 2017). Moreover, it is estimated that annual cases of antimicrobial resistance-induced death will reach 10 million by 2050, which will outpace cancer, diabetes, diarrheal diseases, and road traffic accidents, becoming the top one cause of death (O’Neill, 2016). The innate immune system is the first line of defense, which plays a crucial role in fighting against microbial infections (Miller, 1980). Nevertheless, since the discovery of the first antibiotic, clinicians are increasingly dependent on antimicrobial agents, while ignoring the role of immunity in fighting infections. The escalation of resistance to antimicrobials has forced us to continue to pursue new therapeutics. As outlined by Dr. Arturo Casadevall of the Albert Einstein College of Medicine, we have already entered the third anti-infective era and need to synergistically combine host immune system and active antimicrobials (Casadevall, 2006). In addition to the combination of immunomodulatory agents for the treatment of infections, studies have shown that several antimicrobial agents have regulatory effects on immune system, which can enhance the defense ability against pathogens (Labro, 2012).

Colistin (polymyxin E) belongs to a group of polymyxin antibiotics. It was discovered in the 1940s but abandoned in the clinical practice in the 1970s due to its increasingly reported of nephrotoxicity. However, due to the prevalence of MDR Gram-negative bacteria, including *Acinetobacter baumannii*, *Klebsiella pneumoniae*, and *Pseudomonas aeruginosa*, colistin has been reused as last resort of antibiotic during the last few years (Li et al., 2006). In our previous study, the nematode *Caenorhabditis elegans* are used to screen immune activators, which have potential immune-stimulating effects and can protect nematode against bacterial infections. Colistin has been confirmed to protect the host against infections by a conserved p38/PMK-1 pathway in the intestine, which is independent of its antimicrobial activity. The bacterial burden is not reduced along with the enhanced immune responses mediated by p38/PMK-1, indicating that p38/PMK-1 pathway participates in the development of host tolerance to infections. Since p38/PMK-1-mediated immune responses are quite conserved from plants to mammals, we aimed to evaluate the immunodulatory effects of colistin on macrophages of rats and identify the molecular mechanism responsible for its immunostimulatory activity (Cai et al., 2014).

## Materials and Methods

### Reagents

Colistin (C-4461), lipopolysaccharides (LPS) (L4391), and carboxylate-modified fluorescent microspheres (L4655, 1.0 µm in diameter) were purchased from Sigma, USA. p38 inhibitor (SB203580) was purchased from MedChemExpress (USA). Fetal bovine serum (FBS) was obtained from Gibco (USA), and bovine serum albumin (BSA) was supplied by Roche (Switzerland). RPMI-1640 medium and phosphate buffered saline (PBS) were provided by HyClone (USA). DMSO and thiazolyl blue (MTT) were purchased from AMRESCO (USA); trypsin and penicillin-streptomycin were obtained from TBD Science (China). Phospho-p38 MAP kinase (Thr180/Tyr182) antibody (#9211) was supplied by Cell Signaling (USA). Horse radish peroxidase (HRP)-conjugated goat anti-rabbit IgG secondary antibody was provided by Solarbio (China), and antibody against β-actin was obtained from Thermo Fisher (USA). Enzyme-linked immunosorbent assay (ELISA) kits for various cytokines were purchased from R&D Systems (USA).

### Animals

Male and female clean-grade Sprague-Dawley (SD) rats, weighing 200–250 g, were provided by Laboratory Animal Center, PLA General Hospital (Beijing, China). Rats were routinely housed with free access to food and water. Generous efforts were made to reduce the amount of animals used and minimize animal suffering. All animal procedures and study protocols were approved by the Ethical Committee for the Use of Animals of PLA General Hospital (2017-x3-51).

### Primary Culture of Rat Macrophages

Isolation and culture of rat macrophages were carried out according to a previously described method with some modifications. (Sampaio et al., 2006) The mice were anesthetized with ether and sacrificed by cervical dislocation. The peritoneal cavity was washed with 15 ml cold PBS. After a gentle massage of the abdominal wall for 1–2 min, the peritoneal fluid containing resident macrophages was collected and centrifuged at 1,500 rpm for 5 min at 4°C. The supernatant was discarded, and the total peritoneal cells were re-suspended in RPMI-1640 medium. Trypan blue dye exclusion assay was applied to determine survival rate higher than 95%. Cells were seeded into six-well plates and incubated at 37°C in a humidified atmosphere containing 5% CO_2_. After 4 h, cells were gently washed twice with PBS. Adherent macrophages were cultured in the fresh RPMI-1640 medium at 37°C in a humidified atmosphere containing 5% CO_2_ prior to further analysis.

### Cell Viability Assay

This experiment was divided into nine groups, including seven colistin groups (5, 10, 20, 40, 60, 80, and 100 μg/ml), one negative control group (cell suspension without colistin), and one blank control group (RPMI-1640 complete medium only). Each group was replicated for five times. After 24-h treatment at 37°C in a humidified atmosphere containing 5% CO_2_, the relative viability of macrophages was evaluated by the MTT assay as previously described with some modifications (Gelain and Moreira, 2008). Briefly, macrophages (100 µl) were seeded into 96-well plates at a density of 1.2×10^6^ cells/ml, and then 20 µl MTT solution (5 mg/ml) was added into each well. The cells were maintained at 37°C in a humidified atmosphere containing 5% CO_2_ for 4 h. The culture medium was then discarded, and plates were shaken with DMSO for 10 min. The optical density (OD) of each well was determined at a wavelength of 490 nm. Relative cell viability = [(OD of colistin treatment group-OD of blank control group)/(OD of negative control group-OD of blank control group)] × 100%.

### Cytokine Assay

Rat peritoneal macrophages were seeded into 24-well plates at a density of 1.2 × 10^6^ cells/ml and incubated for 4 h until the cells were stable. The cells were treated with 5, 10, or 20 μg/ml colistin. The same volume of LPS (10 μg/ml) was used as the positive control. After incubation for 6 h, the cell-free supernatants were collected and stored at −20°C until the quantification of cytokines. The concentrations of tumor necrosis factor-α (TNF-α), interleukin-1β (IL-1β), and IL-6 were determined using commercially available ELISA kits according to the manufacturer’s instructions. Automated microplate ELISA reader was applied to measure color development at 450 nm. Standard curve was generated for each assay plate by measuring the absorbencies of serial dilutions of each recombinant cytokine at a wavelength of 450 nm.

### Phagocytosis Assay Against Microspheres

Rat macrophages were seeded onto 12-well plates at a density of 1.2 × 10^6^ cells/well. Cells were exposed to different concentrations (5, 10, or 20 µg/ml) of colistin for 6 h, and 10 µg/ml LPS was employed as the positive control. Phagocytosis assay was applied as previously described with some modifications. (Wang et al., 2015) After the supernatant was discarded, cells were treated with 1 ml carboxylate-modified yellow-green (YG) microsphere (1 μm in diameter) suspension (1:100 in 1% BSA at a concentration of 4.5 × 10^6^ beads/ml) for 2 h at 37°C in dark with gentle shaking to avoid non-specific adhesion and ensure adequate contact. Cells were analyzed by flow cytometry (FC500, Beckman Coulter). A total of 10,000 events were acquired from each run. The percentage of phagocytosis (PP) was defined as: PP = M1 + M2 + M3 + M4, where M1, M2, M3, and M4 refer to the number of cells with one, two, three, and four microspheres, respectively. The relative mean fluorescence intensity (RMFI) was defined as: RMFI = MFI of treatment group/MFI of control group.

### Phagocytosis Assay Pretreated With SB203580 Against Microspheres

Rat macrophages were cultured at a density of 1.2 × 10^6^ cells/well in 12-well microplates for 24 h. Colistin was added as a single dose of 20 µg/ml to cells pretreated with SB203580 (10 µM) for 0.5 h, and LPS was employed as positive control. After an additional 6-h incubation, 1 ml carboxylate-modified yellow-green (YG) microsphere (1 μm in diameter) suspension was administrated for 2 h at 37°C in dark with gentle shaking. Then cells were analyzed by flow cytometry and the method was as described above.

### Phagocytosis Assays Against *Staphylococcus Aureus*

*Staphylococcus aureus* was grown in LB at 37°C for 8 h. The bacterial suspension was centrifuged, and the supernatant was discarded. Then the bacteria were diluted to 7.5 × 10^6^ CFU/ml in saline. Macrophages were seeded into 12-well plates at a density of 1.2 × 10^6^ cells/well and incubated with 20 µg/ml of colistin or 10 µg/ml LPS for 6 h. Subsequently, 30 μl bacterial suspension was added into each well, and the mixture at a ratio of 1:1 was placed on a slide. After incubation at 37°C for 30 min and air dry for 30 min, the slide was stained with Wright-Giemsa for 4 min. Then the slide was gently rinsed with distilled water. Finally, the slide was observed under microscope (Olympus CX31). Phagocytic index was defined as the mean number of associating (binding and uptake) *S. aureus* per phagocytosing cell multiplied by the percentage of phagocytes involved in phagocytosis (Wu et al., 2016). No less than 100 cells were included to quantify the phagocytic index.

### Microarray Assay

Briefly, 1 × 10^6^ macrophages were seeded into each well and incubated in normal culture medium in the absence or presence of 20 μg/ml colistin. The cells were harvested after 3-h exposure. TRIzol reagent (Invitrogen) was added to extract total RNA, followed by a purification process by QIAGEN RNeasy Mini Kit (Qiagen) according to the manufacturer’s instructions. Three independent batches of cells were performed for miRNA extraction and subsequent microarray analysis. Affymetrix GeneChip miRNA 4.0 Array was applied to identify the differentially expressed genes between colistin-stimulated and naive macrophages. The clustered heatmap was visualized using pheatmap. The RVM (random variance model) t-test was applied to filter differentially expressed genes. After the significance analysis and FDR (false discovery rate) analysis, the differentially expressed genes were selected according to a p < 0.05 and a fold change of more than 1.4 between any two groups. The prediction database, miRanda, was used to predict target genes of differentially expressed miRNAs. Finally, pathway analysis was used to determine significant pathways for differentially expressed genes in colistin-stimulated cells using microarray gene pathway annotations downloaded from Kyote Encylopedia of Genes and Genomes (KEGG) (http://www.genome.jp/kegg/). Fisher’s exact test and χ^2^ test were applied to identify the significant KEGG categories, and FDR was used to correct the p values (p < 0.05).

### Western Blotting Analysis

Macrophages were exposed to 5, 10, or 20 μg/ml colistin, and 10 μg/ml LPS was used as the positive control. After incubation for 1 h, stimulated peritoneal macrophages were lysed and subjected to 12% SDS-PAGE. After electrophoresis, proteins were electro-transferred onto polyvinylidene difluoride (PVDF) membranes. The membranes were blocked with 5% skim milk at room temperature for 1 h and then incubated with primary antibody (1:1,000 in 5% skim milk) at 4°C overnight. Membranes were washed for 3 × 5 min with TBST. Subsequently, blots were incubated with HRP-conjugated secondary antibody (1:5,000 in 5% skim milk) for 1 h. Membranes were then washed for 3 × 5 min with TBST again. Finally, immunoreactive bands were visualized by enhanced chemiluminescence (ECL, Thermo Scientific Pierce, USA) according to the manufacturer’s instructions and quantified using a BioSpectrum Imaging System (UVP, CA, USA).

### Statistical Analysis

Results were expressed as means ± SD of three or five independent experiments. Data from the control and treatment groups were analyzed with one-way analysis of variance (ANOVA), followed by Dunnett’s t-test. *P* values less than 0.05 were regarded as statistically significant.

## Results

### Effects of Colistin on the Viability of Macrophages

**Figure 1:**
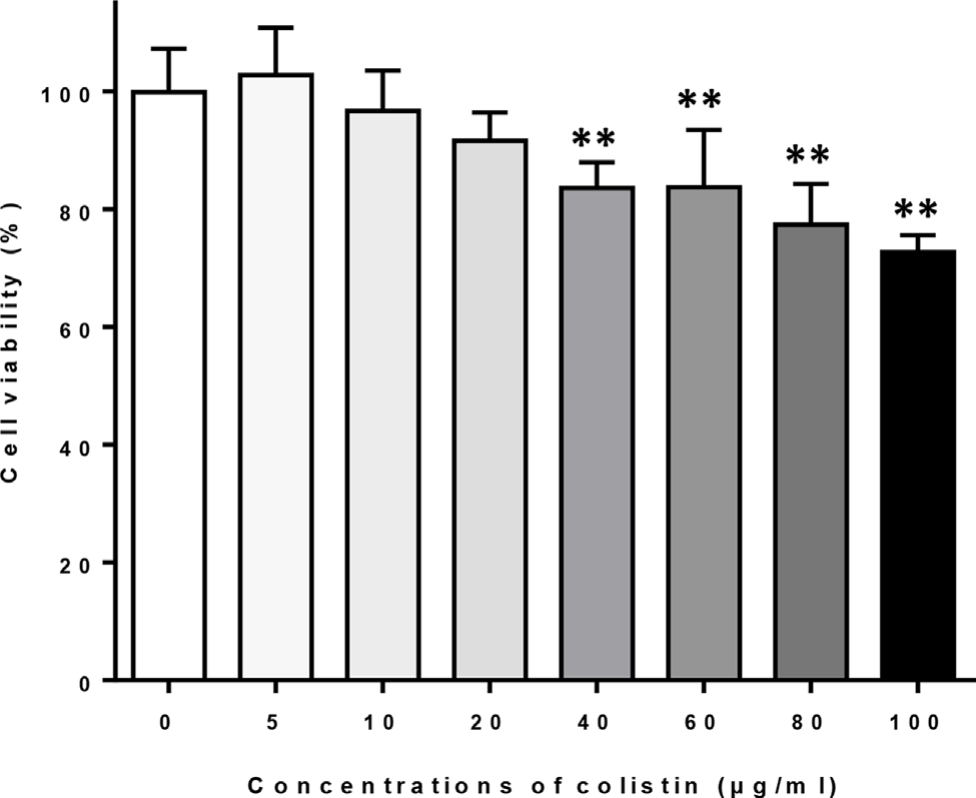
The effects of different concentrations of colistin on the cell viability of macrophages. Data were expressed as mean ± SD of five experiments. **P < 0.01 *versus* control group (colistin = 0).

Macrophages were exposed to different concentrations of colistin for 24 h, and the cell viability was evaluated by MTT assay. **Figure 1** shows that exposure of colistin at concentrations of 5, 10, and 20 μg/ml did not affect the cell viability, whereas higher concentrations of colistin (40, 60, 80, and 100 μg/ml) significantly reduced the viability of cells. Therefore, 5, 10, and 20 μg/ml of colistin were used in subsequent experiments.

### Effects of Colistin on Release of Cytokines TNF-α, IL-6, and IL-1β

**Figure 2:**
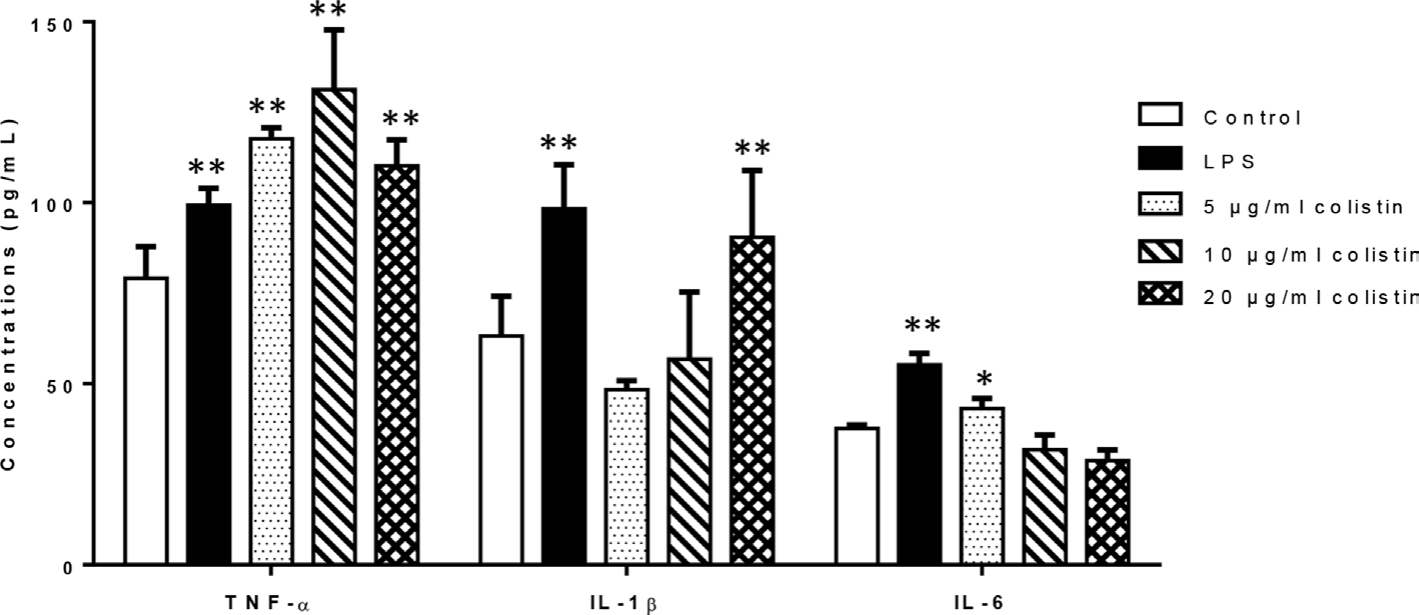
The effect of colistin on the secretion of cytokines TNF-α, IL-6, and IL-1β after 6 h of treatment. Data were expressed as mean ± SD of three experiments. *represents *P* < 0.05 *versus* control group, **P < 0.01 *versus* control group.

We investigated the effects of colistin on release of cytokines (TNF-α, IL-6, and IL-1β). **Figure 2** illustrates that after 6-h treatment of colistin at 5, 10, and 20 µg/ml, the secretion of TNF-α in colistin treatment groups was significantly increased compared with the control as positive control (LPS) group did. In case of IL-1β, only 20 µg/ml colistin significantly increased the secretion of IL-1β. In addition, 5 µg/ml of colistin increased the IL-6 secretion, showing the same effect as LPS, while 10 and 20 µg/ml colistin did not change the IL-6 secretion.

### Colistin Increases Phagocytosis of Macrophages Through p38/MAPK Pathway

**Figure 3:**
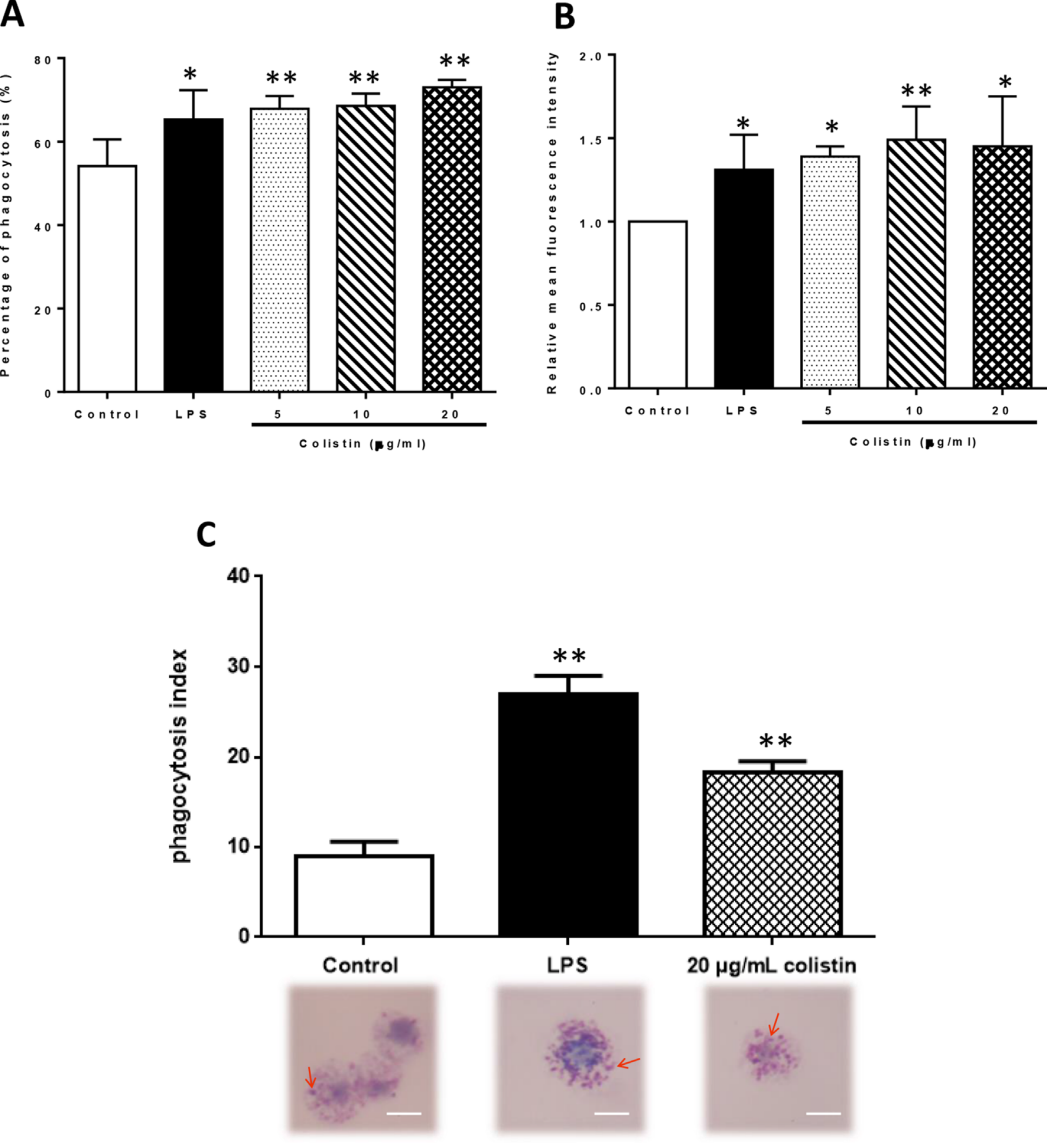
The effect of colistin on phagocytotic ability of macrophages after 6 h of treatment.** (A)** Percentage of phagocytosis (PP) of macrophages against microspheres. Data were expressed as mean ± SD of three experiments. **(B)** Relative mean fluorescence intensity (RMFI) of microspheres. Data were expressed as mean ± SD of three experiments. **(C)** Phagocytic index against *S. aureus*. *S. aureus* was indicated by red arrows. Scale bars: 5 μm. Data were expressed as mean ± SD of twenty cells which were counted. *P < 0.05 *versus* control group, **P < 0.01 *versus* control group.

After the exposure to 5, 10, and 20 µg/ml colistin, the phagocytic ability of macrophages against microspheres was improved in all colistin treatment groups. Flow cytometry revealed that the PP in colistin treatment groups was significantly higher compared with the control group (p < 0.05), reaching the level of LPS group (**Figure 3A**). However, the RMFI also showed the same trend in colistin treatment groups (**Figure 3B**). The phagocytic ability of macrophages against *S. aureus* was also significantly increased after colistin treatment. Phagocytic index was greatly increased in 20 µg/ml colistin treatment group compared with the control group (p < 0.01, **Figure 3C**).

**Figure 4:**
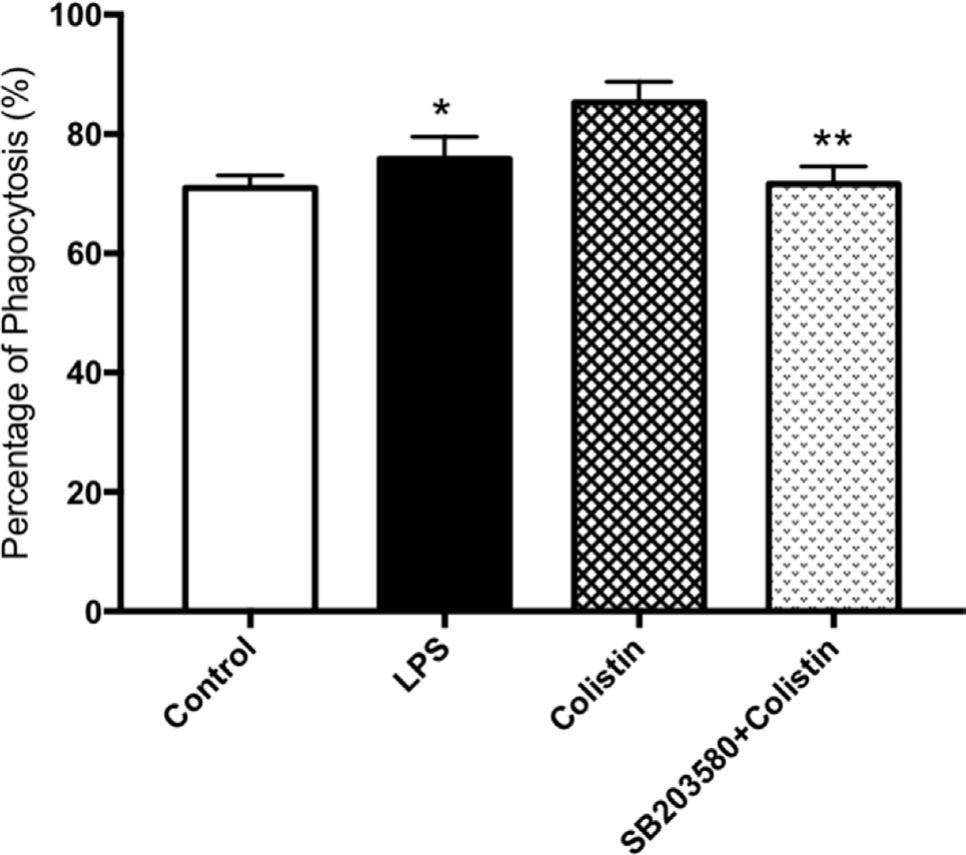
Colistin increases the phagocytosis of macrophages through p38/MAPK pathway. PP of macrophages against microspheres. Data were expressed as mean ± SD of three experiments. *P < 0.05 *versus* colistin group, **P < 0.01 *versus* colistin group.

Cells pre-incubated with SB203580 have showed lower phagocytic ability against microspheres. Flow cytometry showed that the PP in the presence of SB203580 group was remarkably lower than those in the absence of SB203580 group (p <0.05, **Figure 4**).

### Differential Gene Expression in Colistin-Stimulated Macrophages

**Figure 5:**
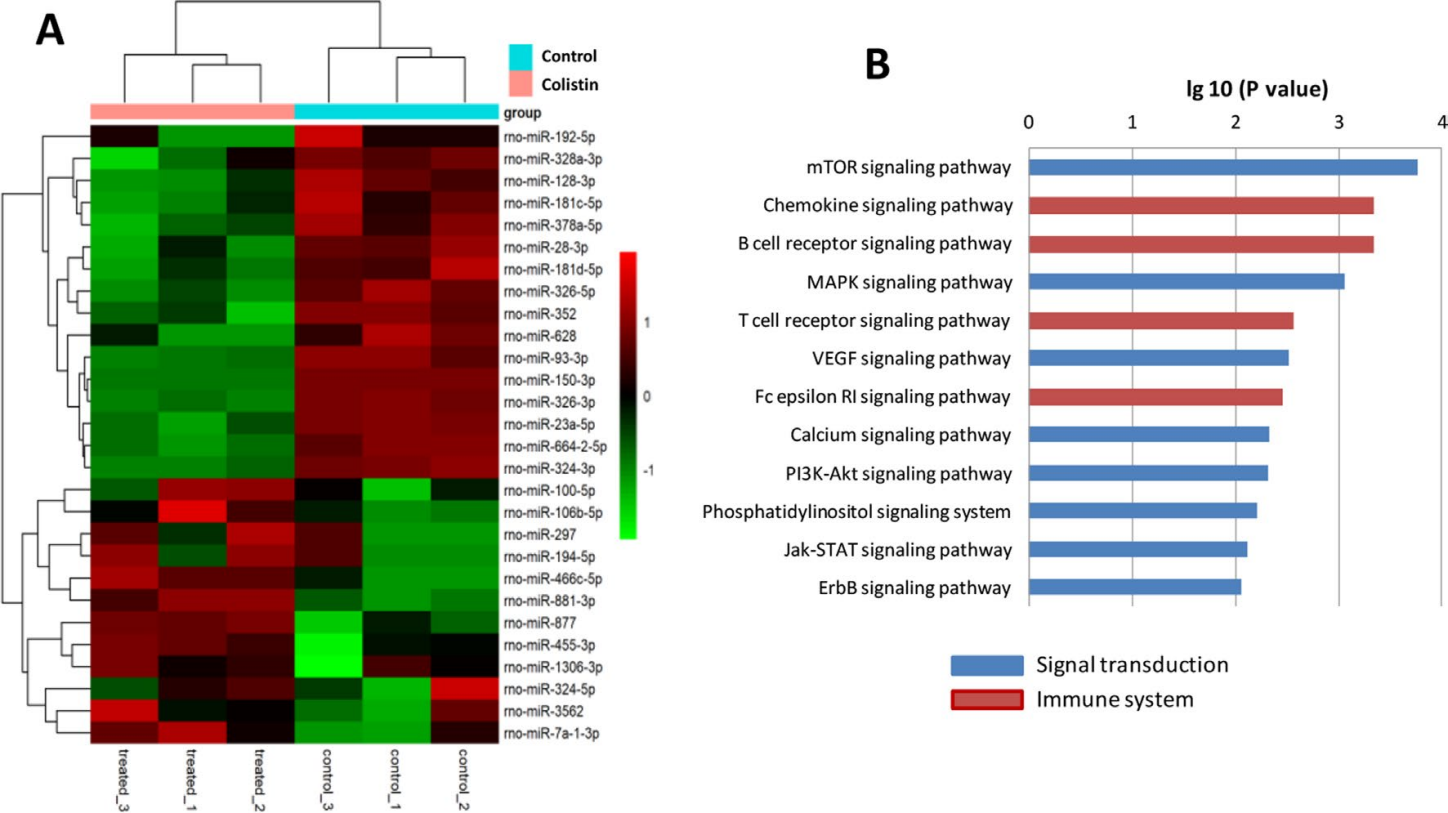
Colistin acts as a general immune activator that elicits an expression profile. **(A)** Microarray results showed that 28 miRNAs were significantly regulated in macrophages, including 16 up-regulated and 12 down-regulated genes in response to colistin treatment. Patterns were plotted on the heatmap using pheatmap. Red represents up-regulated genes, while green represents down-regulated genes. Hierarchical clustering is shown on the left. **(B)** KEGG pathway analysis showed the significantly increased genes set linked to immune or signal transduction.

Microarray results showed that the expressions of 28 miRNAs in macrophages were significantly altered after colistin challenge (**Figure 5**). A total of 549 target genes were predicted for these differentially expressed miRNAs by miRanda database (**Table S1**). KEGG pathway analysis revealed that the dysregulated miRNAs regulated several environmental information processing and immune system-related pathways, such as the mitogen-activated protein kinase (MAPK) signaling pathway, mammalian target of rapamycin (mTOR) signaling pathway, chemokine signaling pathway, and B cell receptor signaling pathway.

### Colistin Increases the Expression of Phosphorylated p38 in Macrophages

**Figure 6:**
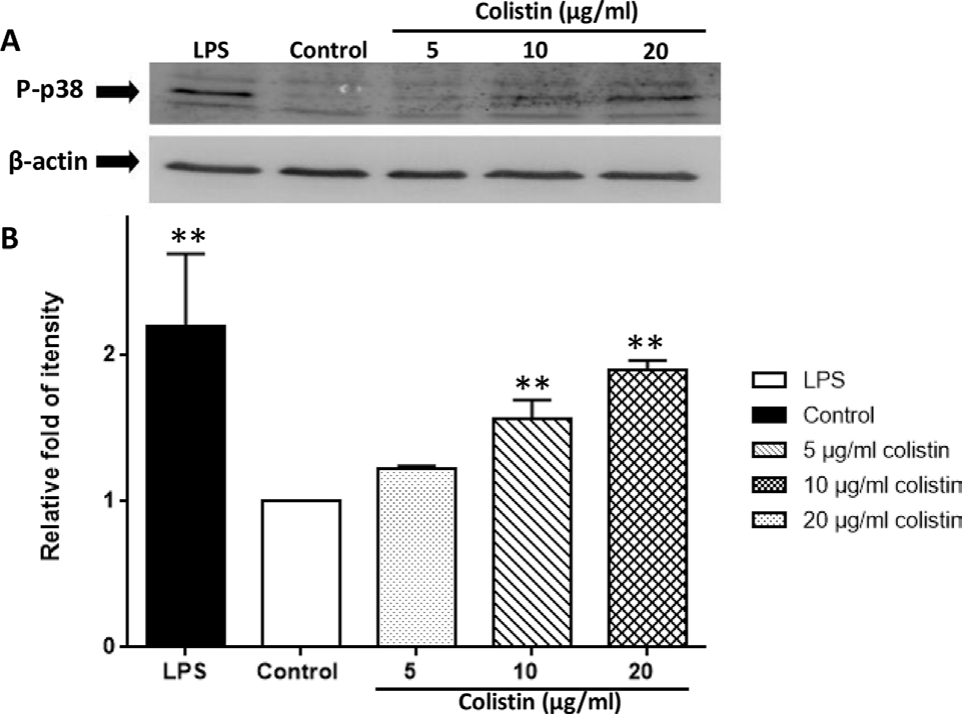
Colistin increases the expression of phosphorylated p38 protein in macrophages. **(A)** Protein expression detected by Western blotting. **(B)** Relative fold as compared with Teh control group. Data were expressed as mean ± SD of three experiments. **P < 0.01 *versus* control group.

**Figure 6** demonstrates that the phosphorylated p38 protein level of 10 and 20 µg/ml colistin treatment groups was significantly higher compared with the control group (p < 0.01). Although the difference was not significant, the phosphorylated p38 protein level of 5 µg/ml colistin treatment group was also numerically higher than that of the control group. Moreover, the phosphorylation of p38 protein occurred in a colistin concentration-dependent manner. The higher concentration of colistin, the higher level of phosphorylated p38 protein.

## Discussion

Nowadays, the exploration on immunological activity of antimicrobial agents has been exemplified by many successes (Ruh et al., 2017). For example, anti-inflammatory and immunomodulatory properties of macrolide antibiotics have been applied in patients with chronic airways diseases (Ratjen et al., 2012; Wong et al., 2012). Colistin is the last choice for multidrug-resistant Gram-negative strains because there are no other effective antimicrobials. However, the resistant reports and the nephrotoxicity of colistin are always a clinical concern (Li et al., 2006; Spapen et al., 2011). Our study found that colistin has the ability of immunomodulation in the early stage. The results may provide preliminary data not only for describing the whole picture of colistin in anti-infective treatment, but also for the possibility of expanding treatment indications of colistin. A comprehensive understanding of an antimicrobial drug is important for its protection and more rational application.

In the present study, we showed that colistin increased the secretion of pro-inflammatory cytokines, including TNF-α, IL-1β, and IL-6. Colistin also greatly promoted the phagocytic ability of macrophages. Furthermore, our study suggested that these immunomodulatory effects appeared to involve several pathways related to immune system and signal transduction, including p38/MAPK pathway, which has been confirmed as a conserved p38/PMK-1 pathway in *C. elegans* in our previous study (Cai et al., 2014). Inflammatory cytokines like TNF-α, IL-1β, and inflammatory stimuli like LPS can activate p38 in macrophages (Shi et al., 2015). In our study, we took LPS as positive control groups. The other experimental factors were strictly controlled the same as negative control groups. Macrophages after treating with colistin had shown an increasing secretion of cytokines TNF-α, IL-6, and IL-1β, an increasing phagocytosis and an increasing phosphorylated p38. Meanwhile, macrophages pretreated with p38 inhibitor did not show an increasing phagocytosis after colistin treatment. Based on above results, we suggest that colistin can stimulate the immunomodulatory effects of rat macrophages by activating the p38/MAPK pathway.

Cytokines play a key role in regulating the immune response during the infection. Cytokines can be divided into pro-inflammatory and anti-inflammatory cytokines (Schulte et al., 2013). Pro-inflammatory cytokines, such as TNF-α, IL-1β, and IL-6, can activate the secondary effect or cells involved in innate or adaptive immune response and trigger a beneficial inflammatory response, such as increased local coagulation and restricted tissue damage (Chaudhry et al., 2013). Zhong et al. (2008) have reported that polymyxin B and colistin are capable of directly activating murine NK cells, one of the primary effector cells of the innate immune system. Both 24-h treatment of 300 μg/ml polymyxin B and colistin can markedly enhance the production of IFN-γ. However, only polymyxin B can increase the level of TNF-α. Macrophages are also the primary cells of the innate immune system, which can produce pro-inflammatory cytokines (Arango Duque and Descoteaux, 2014). To the best of our knowledge, we, for the first time, demonstrated that colistin could stimulate the secretion of pro-inflammatory cytokines in macrophages in a short term (3 and 6 h, data of 3 h not shown here). This cytokine-stimulating effect of colistin should be directly exerted on macrophages. These results were also consistent with our previous report that the direct exposure of the intestinal cells of the *C. elegans* to 20 μg/ml of colistin for 24 h appears to allow colistin to reach intracellular concentrations appropriate to activate immune pathways and confer resistance to pathogen infections (Cai et al., 2014). Colistin has also been reported to decrease the secretion of cytokines induced by LPS. Matzneller et al. (2017) have performed an *ex vivo* study and a randomized controlled trial (RCT) to evaluate colistin-induced modulatory effect on inflammatory response in LPS-challenged human endotoxemia model. *Ex vivo* study has shown that the expressions of TNF-α, IL-1β, IL-8, and IL-6 induced by LPS in venous blood are significantly reduced after 4 h of incubation with colistin. RCT results have also confirmed that the response of inflammatory cytokines (IL-6, IL-8, and TNF-α) to LPS is significantly decreased after colistin treatment. However, because observed decrease of cytokines is consistent with the reduction of LPS level in circulation of volunteers, they speculate that colistin exerts its cytokine-reducing effect through its endotoxin-neutralizing property by binding LPS, instead of direct action on cells.

Cells of the innate immune system can remove the invading microorganisms and other foreign particles by phagocytosis. Therefore, the enhancement of phagocytosis is also a hallmark of the activation of macrophage’s immune function (Huynh et al., 2007). Our study clearly showed that colistin treatment could greatly improve the phagocytic ability of macrophages against both microspheres and *S. aureus*. Moreover, the phagocytosis of macrophages decreased significantly after pretreating with p38 inhibitors, suggesting that colistin stimulated macrophage phagocytosis through p38/MAPK pathway. The enhanced phagocytic ability to *S. aureus* was not related to the antibacterial activity of colistin, because *S. aureus* is naturally resistant to colistin and macrophages were washed after colistin treatment and then incubated with *S. aureus*. Together with its cytokine-stimulating effect, colistin exerted immune activating effect on macrophages.

Many genes are altered in the course of enhancement of macrophage’s immune function. Our study results showed that 16 miRNAs up-regulated and 12 miRNAs down-regulated in macrophages after colistin treatment. For example, Dusp7 encoded by rno-miR-297 was upregulated significantly. Dusp7 is a member of the DUSPs family which can combine with p38 and regulate the activity of MAPK pathway (Levy-Nissenbaum et al., 2003). According to our previous *C. elegans* experiment, colistin-triggered activation of the innate immunity is p38/PMK-1-dependent (Cai et al., 2014). In the present study, results from microarray and Western blotting analysis further confirmed that p38/MAPK signaling pathway was at least one of the key pathways that might involve in colistin’s immunomodulatory effect in mammals. Similar effect of colistin on MAPK pathway has only been reported in colistin-induced nephrotoxicity. Dai et al. (2014) have observed that the expressions of MAPK family markers, including p-p38, are significantly enhanced in kidney tissue of rats in a dose-dependent manner after colistin treatment.

Autophagy is one of the fundamental eukaryotic pathways, which is immunity related. Moreover, it acts as original form of innate immunity against invading microorganisms (Deretic et al., 2013). As an important housekeeping mechanism that rapidly responds to environmental stresses, autophagy plays a critical role in both innate and adaptive immunities (Gomes and Dikic, 2014). Our study revealed that the autophagy might participate in the immunomodulatory effect of colistin, as the mTOR and P13K-Akt signaling pathways were significantly activated during the colistin treatment, which are generally known to involve in the autophagic process. Colistin-induced autophagy has been only studied in PC-12 cells, indicating that it may protect neurons by reducing the colistin-induced cytotoxicity (Zhang et al., 2015; Lu et al., 2017a; Lu et al., 2017b).

Taken together, present study is the first to demonstrate that colistin had the immunomodulatory effect on macrophages in mammals, and the p38/MAPK pathway was involved in colistin-induced immunomodulatory effect. From a previous study, the C_max_ of colistin measured 2.33 ± 0.16 mg/L in rats plasma after an intravenous bolus of 1 mg/kg (Li et al., 2003). Though we have chosen a high concentration of colistin used in our study (5, 10, and 20 μg/ml) and in the instructions of colistin sulfate for injection, the C_max_ of colistin after intravenous injection was 2–3 μg/ml, it has been widely recognized that drug concentrations are remarkably different between *in vivo* and *in vitro* studies (Dai et al., 2013). Our future studies might focus on this effect *in vivo*. Meanwhile, comprehensive elucidation of the signaling pathways responsible for colistin-induced immunomodulatory effect and their interplay is also warranted. The study of the immunomodulatory mechanism of colistin not only helps us better understand the effectiveness and safety of colistin in the treatment of infections, but also more likely to broaden its clinical usage beyond its antimicrobial activity.

## Ethics Statement

Male and female clean-grade Sprague-Dawley (SD) rats, weighing 200–250 g, were provided by Laboratory Animal Center, PLA General Hospital (Beijing, China). Rats were routinely housed with free access to food and water. Generous efforts were made to reduce the amount of animals used and minimize animal suffering. All animal procedures and study protocols were approved by the Ethical Committee for the Use of Animals of PLA General Hospital (2017-x3-51).

## Author Contributions

JW, WS, HN and TY contributed to the laboratory data acquisition, data analysis, and drafting of article. YW contributed to the design of the study and critical revision. YC contributed to the conception and design of the study, analysis of data, and drafting of the article and critical revision.

## Funding

This research was supported by the National Natural Science Foundation of China (No. 81573472 and No. 81770004).

## Conflict of Interest Statement

The authors declare that the research was conducted in the absence of any commercial or financial relationships that could be construed as a potential conflict of interest.
